# Inhibiting Extracellular Vesicle Release from Human Cardiosphere Derived Cells with Lentiviral Knockdown of nSMase2 Differentially Effects Proliferation and Apoptosis in Cardiomyocytes, Fibroblasts and Endothelial Cells *In Vitro*

**DOI:** 10.1371/journal.pone.0165926

**Published:** 2016-11-02

**Authors:** Jennifer K. Lang, Rebeccah F. Young, Hashmat Ashraf, John M. Canty

**Affiliations:** 1 Department of Medicine, Division of Cardiology, Jacobs School of Medicine and Biomedical Sciences, Buffalo, N.Y, 14203, United States of America; 2 Department of Cardiothoracic Surgery, Kaleida Health, Buffalo, N.Y, 14203, United States of America; 3 VA WNY Healthcare System, Buffalo, N.Y., 14215, United States of America; Georgia Regents University, UNITED STATES

## Abstract

Numerous studies have shown a beneficial effect of cardiosphere-derived cell (CDC) therapy on regeneration of injured myocardium. Paracrine signaling by CDC secreted exosomes may contribute to improved cardiac function. However, it has not yet been demonstrated by a genetic approach that exosome release contributes to the therapeutic effect of transplanted CDCs. By employing a lentiviral knockdown (KD) strategy against neutral spingomyelinase 2 (nSMase2), a crucial gene in exosome secretion, we have defined the role of physiologically secreted human CDC-derived exosomes on cardiac fibroblast, endothelial cell and primary cardiomyocyte proliferation, cell death, migration and angiogenesis using a series of *in vitro* coculture assays. We found that secretion of hCDC-derived exosomes was effectively inhibited by nSMase2 lentiviral KD and shRNAi expression was stable and constitutive. hCDC exosome release contributed to the angiogenic and pro-migratory effects of hCDCs on HUVECs, decreased proliferation of fibroblasts, and decreased apoptosis of cardiomyocytes. These *in vitro* reactions support a role for exosome secretion as a paracrine mechanism of stem cell-mediated cardiac repair *in vivo*. Importantly, we have established a novel tool to test constitutive inhibition of exosome secretion in stem cell populations in animal models of cardiac disease.

## Introduction

In the last decade, experimental and clinical studies have shown a beneficial effect of cell therapy on left ventricular (LV) function in disease states associated with myocyte loss such as cardiomyopathy (ischemic and non-ischemic), myocardial infarction and hibernating myocardium. Interestingly, regardless of the platform used, there appears to be a disproportionate benefit of cellular therapy on functional improvement in relation to the number of injected stem cells that remain in the heart or transdifferentiate into cardiac myocytes in large animal models of cardiovascular disease [1–5]. This has given rise to the notion that many therapies work through a paracrine signaling mechanism that stimulates endogenous myocyte and capillary formation while at the same time, inhibiting fibrosis [6–9].

Cardiosphere-derived cells (CDCs) have been advanced to clinical trials as a platform for cardiopoietic cell therapy. They have been shown to decrease scar size, increase viable myocardium, and improve regional function of infarcted myocardium at 1 year post-treatment in the phase 1/2 CArdiosphere-Derived aUtologous StemCElls to reverse ventricUlar dySfunction (CADUCEUS) clinical trial[10]. While they stimulate regeneration, angiogenesis, and functional improvement in infarcted myocardium [11, 12], these benefits likely occur by indirect mechanisms. This conclusion is based on the fact that most of the newly regenerated myocardium and vasculature are from cells of endogenous origin [2, 13] and the beneficial effects of CDCs persist long after the injected cells have been cleared by the host [14]. Thus, paracrine factors released by CDCs have become candidates for effecting cardiac repair.

Recent reports indicate that extracellular membrane vesicles participate in paracrine signaling, including the transfer of functional proteins, mRNAs and microRNAs. Exosomes are bi-lipid membrane vesicles with diameters of 40-100nm that originate in the late endosomal compartment within intracellular multivesicular bodies (MVBs). After fusion of MVBs with the plasma membrane, pools of exosomes are released into the extracellular environment and function as vectors of cell to cell communication. In support of their role in preventing postinfarcrion LV remodeling, injection of a large number of highly purified stem cell-derived exosomes directly into the myocardium reduced infarct size and prevented the decline in systolic function due to left ventricular remodeling following myocardial infarction [15–19]. Developing an approach to inhibit exosome release from stem cells on a chronic basis is required to dissociate their importance from paracrine factors released in an exosome independent manner.

Neutral sphingomyelinase 2 (nSMase2) has been shown to contribute to exosome secretion by triggering the budding of exosomes into MVBs [20], and shRNA blockade of nSMase2 inhibits the release of exosomes from human macrophages (THP-1 cells) [21]. In models of myocardial infarction, the contribution of paracrine secreted CDC exosomes to functional improvement of the heart, their mechanism of uptake, and how this may be targeted remain important parameters. Accordingly, we have taken a genetic approach to selectively block exosome release by shRNA KD of nSMase2. Our findings support a role of exosomes on endogenous cardiac repair but demonstrate a divergent effect among specific resident cardiac cell types.

## Materials and Methods

### Human CDC Culture

Following written informed consent, endomyocardial biopsies from the right atrial appendage were obtained from four adult patients undergoing on pump CABG at Buffalo General Medical Center under approved Institutional Review Board protocols. Cardiosphere-derived cells were grown as previously described from each patient [2]. In brief, heart biopsies were minced into small fragments, washed, and briefly digested with type IV collagenase for 60 minutes at 37°C. Explants were then cultured on 20μg/ml fibronectin-coated dishes. A layer of stromal-like cells and population of small, round, phase-bright cells migrated out to surround the explants. Once confluent, the cells surrounding the explants were harvested by gentle enzymatic digestion. These cardiosphere-forming cells were seeded at ~ 1×10^5^ cells/mL on low attachment dishes in cardiosphere medium (20% heat-inactivated fetal calf serum, pen/strep 100 μg/ml, 2 mmol/L L-glutamine, and 0.1 mmol/L 2-mercaptoethanol in Iscove’s modified Dulbecco medium). Cardiospheres formed and began to slowly grow in suspension. After 4–6 days in culture, when sufficient in size and number, free-floating cardiospheres were harvested by aspirating them along with media. Detached cardiospheres were then plated on fibronectin-coated flasks where they attached to the culture surface and formed monolayers of “Cardiosphere-Derived Cells” (CDCs) [11]. CDCs were subsequently passaged by trypsinization and splitting at a 1:8 ratio. Up to 100 million CDCs developed within 4–6 weeks of the time that the original cardiac biopsies were obtained. Cells were characterized by flow cytometry and immunohistochemistry with hematopoietic (CD45, cKit, CD133), mesenchymal (CD90, CD105) and cardiac (GATA4, Nkx2.5, and cTnI) markers.

### Human Cardiac Fibroblast Culture

Cardiac fibroblasts were isolated from right atrial appendage endomycoardial tissue biopsies obtained as described above. Cells were isolated and cultured according to previously described methods [22]. Discoidin domain receptor (DDR)2 (mouse monoclonal, Abcam), a collagen receptor shown to be expressed specifically in cardiac fibroblasts but not myocytes, endothelial cells or vascular smooth muscle cells, was chosen for ICC validation of our primary cell line [23–25] and cultures showed near 100% purity.

### Mouse Primary Cardiomyocyte Culture

All animal experiments were performed according to protocol approved by the University at Buffalo Institutional Animal Care and Use Committee. Day 1–3 neonatal mice were euthanized by cervical dislocation and their hearts were dissected. Tissue digestion was performed using the Pierce Primary Cardiomyocyte Isolation Kit (ThermoScientific). For each experiment, dissociated cardiomyocytes were pooled from 10 neonatal mouse hearts (total yield approximated 12x10^6^ cells). Isolation and plating of cardiomyocytes was accomplished within two hours of animal euthanization to obtain high cell yield and reproducible viability. Cells were plated at 4.0 x 10^4^ cells per 96 well or 5.0 x 10^5^ per 24 well. Cardiomyocyte purity was over 80% at one week with strong synchronous contractions of cells in the wells visualized by phase contrast microscopy after 3 days in culture.

### Immunocytochemistry and Flow Cytometry Characterization

CDCs were dissociated to single cell suspension by incubation with 0.05% Trypsin-EDTA and resuspended in DMEM/F12 with HEPES (Gibco) supplemented with 10% FBS (FACS Staining Medium). They were incubated with conjugated antibody at 1:10 dilution for 1 h at 4°C prior flow cytometry analysis (BD FACSCalibur). Antibodies used include CD105 (mouse monoclonal, Abcam), CD90 (mouse monocolonal, BioLegend), CD45 (mouse monocolonal, AbD Serotec), mouse IgG1-FITC (R&D Systems), and mouse IgG1-APC (Miltenyi Biotec). For immunocytochemistry, cells were fixed with 4% paraformaldehyde and immunostained with primary antibodies GATA4 (goat polyclonal, R&D Systems), Nkx2.5 (goat polyclonal, R&D Systems), and cardiac troponin I (mouse monoclonal, Abcam). Primary antibodies were used at a 1:100 dilution. Alexa Fluor conjugated 488 or 555 donkey anti-mouse, goat or rabbit secondary antibodies (Molecular Probes) were added at a dilution of 1:500 for 1 hour at room temperature. Fixed cultures were counterstained with DAPI (Sigma). Images were obtained using a Zeiss Axiovert 200 inverted fluorescence microscope.

### shRNAi Lentiviral Generation and Creation of hCDC nSMase2 Knockdown Line

shRNA targeting human nSMase2 (National Center for Biotechnology Information Nucleotide database accession code NM_018667) constructed with the lentiviral vector pLKO.1 (Addgene), and the scrambled shRNA LV control vector, were generously provided by Dr. Zhenghong Yuan [21]. We subsequently generated nSMase2 and scrambled lentiviruses using triple transfection of the lentiviral backbone with helper plasmids pLP/VSVG (Invitrogen) and psPAX2 (AddGene, #12260) into 293T cells using Fugene HD (Promega). Viral supernatants were collected in OPTI-MEM (Invitrogen) at 48 and 72 hours and yielded a titer of at least 10^6^ GFP-transducing U/ml. Human CDCs were transduced at 1 MOI in the presence of polybrene (Sigma) and stable lines selected with puromycin (Sigma) at a concentration of 2μg/ml for 72 hours. Confirmation of nSMase2 knockdown was confirmed at P4, corresponding to the passage at which all experiments (exosome production and co-culture assays) were performed.

### Media Conditioning and Exosome Purification

Similarly to previously described methods by other groups, exosomes were harvested from CDCs at P4 [15]. This passage also corresponds to the passage of CDCs transplanted into our porcine model of hibernating myocardium [1, 2]. As a control, we also isolated exosomes from normal human dermal fibroblasts (NHDF) at P4. 1.3 x 10^6^ CDCs and NHDFs were conditioned in media containing exosome free serum (System Biosciences) for 48 hours in T75 flasks. Media was then centrifuged at 3,000xg for 15 minutes to remove cellular debris. Exosomes were then isolated using either differential centrifugation by previously described methods [26] or Exoquick Exosome Precipitation Solution [27] (System Biosciences), which both yielded high quantities of purified exosomes. Exosome pellets were then resuspended in the appropriate media and used for assays. Protein content of exosomes were quantified using a Pierce BCA Protein Assay Kit (Thermo Scientific).

### Transmission Electron Microscopy

Exosome pellets were isolated and samples prepared by negative staining method using uranyl acetate. A 20ul aliquot was fixed with 4% PFA for 30 minutes; 6ul was then absorbed onto 200 mesh Formar-carbon coated EM grids (Electron Microscopy Sciences) for 2 minutes and the excess blotted on filter paper. Grids were allowed to dry for 20 minutes then washed twice with MilliQ water and negatively stained with 2.5% uranyl acetate. Data were collected on a JEOL 100CX transmission electron microscope operating at 150kV.

### Particle Analysis

Exosome pellets were suspended in 1mL of MilliQ water, gently vortexed at 2.5k for 10 sec and then bath sonicated for 10 min at 33°C to ensure adequate exosome dispersion in the solution. Particle analysis data was obtained using an LM10 NanoSight instrument.

### Immunoblot analysis

Immunoblot analyses were done with the appropriate antibodies **(Table 1)** according to standard protocols.

**Table 1:** Antibodies used for immunoblot analysis.

Antibody name	Company	Product #	Concentration
Mouse anti-Human nSMase2 (SMPD3)	R&D Systems	MAB7184	1 μg/mL
Mouse anti-Human/Mouse/Rat EEA1	R&D Systems	MAB8047	1.5 μg/mL
Mouse anti-Human CD63	Santa Cruz	sc-5275	1 μg/mL
Mouse anti-Human/Mouse/Rat Cytochrome C	R&D Systems	MAB897	0.5 μg/mL
Goat anti-Human/Mouse/Rat HSP90	R&D Systems	AF3775	0.5 μg/mL
Rabbit anti-Human/Mouse/Rat/Monkey/Pig/Cow GAPDH	Cell Signaling Technology	2118	1:1000
Rabbit anti- Human/Mouse/Rat/Monkey Grp94	Cell Signaling Technology	2104	1:1000

### Cellular Uptake of Exosomes

Exosomes were isolated from conditioned media of cells at P4 as described above and resuspended in 500ul of 1x PBS. 50ul of 10x Exo-Red (Acridine Orange chemistry) or Exo-Green (Carboxyfluorescein succinimidyl diacetate ester (CFSE) chemistry) (System Biosciences) was added to 500ul of resuspended exosomes suspension, inverted to mix, and incubated at 37°C for 10 minutes. To stop the reaction, 100ul of ExoQuick-TC was added (System Biosciences) and the suspension was mixed by inverting 6 times. The labeled exosome sample was placed on ice for 30 minutes then centrifuged for 3 minutes at 14,000rpm. The supernatant was removed and the labeled exosome pellet was resuspended in 500ul of 1x PBS. Target cells were incubated with labeled exosomes and their uptake assessed by flow cytometry (BD FACSCalibur) and confocal microscopy (Zeiss LSM 510 Meta NLO Confocal Microscope) at various time points.

### Endothelial Migration Assay

HUVECs grown on collagen coated TC plates were cocultured with either media control alone, Scr CDCs, or nSMase2 KD CDCs grown on 0.4 μm pore polyester transwell membrane inserts (Corning) for 24 hours. They were subsequently trypsinized and seeded at a density of 4 x 10^4^ cells per insert in 24 well 8.0 μm pore inserts (Corning) coated with collagen. Cells were maintained in EBM2 supplemented with an additional 10% of exosome free serum for 4 h at 37°C and 5% CO_2_ prior to fixation in 4% PFA, counterstaining with DAPI, and quantification of total cells and migrated cells (after removal of non-migrated cells from the upper surface of the transwell membrane by “scrubbing”, performed by inserting a cotton tipped swab into the inserts and applying gentle but firm pressure while moving the tip over the membrane surface). Five fields per well were imaged with a Cellomics High Content Screening device at 20x magnification. Experiments were performed in triplicate using CDCs at P4. Significant differences in percentage of migrating cells with each coculture condition was determined using a one-way ANOVA with a Tukey’s post-hoc test, p<0.05.

### Matrigel Tube Formation Assay

HUVECs cocultured with either media control alone, Scr CDCs or nSMase2 KD CDCs on transwell inserts (0.4μm Pore Polyester Membrane Insert, Corning) for 24 hours were trypsinized and seeded at a density of 5 × 10^3^ cells per well in a 96 well tissue culture plate coated with 50ul of growth factor depleted Matrigel (BD Biosciences). Cells were maintained in EBM2 supplemented with an additional 10% of exosome free FBS and 50ng/ml VEGF at 37°C and 5% CO_2_. After incubating for 4h, tube formation by endothelial cells was photographed (Nikon inverted microscope, 4x objective) and quantified by measuring the length of tubes with NIH Image and counting the branch points in 10 random fields. Experiments were performed in triplicate using CDCs at P4. Significant differences in vessel length and number of branch points with each treatment was determined using a one-way ANOVA with a Tukey’s post-hoc test, p<0.05.

### BrdU Proliferation Assay

HUVECs, cardiac fibroblasts and mouse neonatal cardiomyocytes were cocultured with either media control alone, Scr CDCs (P4), or nSMase2 CDCs (P4) on 0.4μm inserts (Corning) for 24 hours in their respective basal media (supplemented with exosome free serum as needed). Cells were pulsed with BrdU (Sigma) for various cell type specific time points (4 hours for HUVECs and cardiac fibroblasts, 5 days for mCM) prior to a 15 minute fixation in 4% PFA, 1 hour incubation with anti-BrdU primary Ab at 1:1000 (rat, Serotec), 1 hour incubation in Alexa Fluor conjugated 488 or 555 goat anti-rat secondary antibody at 1:500 (Molecular Probes), and counterstaining with DAPI.

### Quantification of cardiomyocyte apoptosis by TUNEL

Freshly dissociated mCM were coculutred with either media control alone, Scr CDCs (P4) or nSMase2 KD CDCs (P4) for 24 hours prior to fixation and detection of myocyte apoptosis by terminal deoxynucleotidyl transferase–mediated dUTP nick end-labeling (TUNEL, Chemicon Inc) and epifluorescence with an FITC filter as described previously[28]. Cultures were costained with TnI and DAPI for quantification.

### TGF-β Stimulation

Human cardiac fibroblasts were treated with reduced serum media to induce cell cycle arrest for 12 hours and subsequently cocultured with either Scr CDCs (P4) or nSMase2KD CDCs (P4) for 24 hours before the addition of TGF-β (10 ng/ml final concentration) for 12 hours. Cell mRNA was isolated with Qiagen miRNeasy Mini Kit according to manufacturer’s instructions. cDNA was generated (M-MLV RT, Promega) and matching quantities of mRNA were reverse transcribed (SYBR Green, Invitrogen) to evaluate CT levels. Relative expression was calculated using the comparative CT method, with GAPDH as the housekeeping gene. The mean minimal cycle threshold values were calculated from triplicate reactions. The following primers were used: COLI (collagen I) F: 5-CCTCAAGGCTCCAACGAG-3’, R: 5’-CAATCACTGTCTTGCCCCA-3’; COLIII (collagen III), F: 5’-TGGTGTTGGAGCCGCTGCCA-3’,R: 5’-CTCAGCACTAGAATCTGTCC -3’.

## Results

### Human CDCs secrete exosomes which are internalized by endothelial cells, cardiac fibroblasts and myocytes

**Fig 1:**
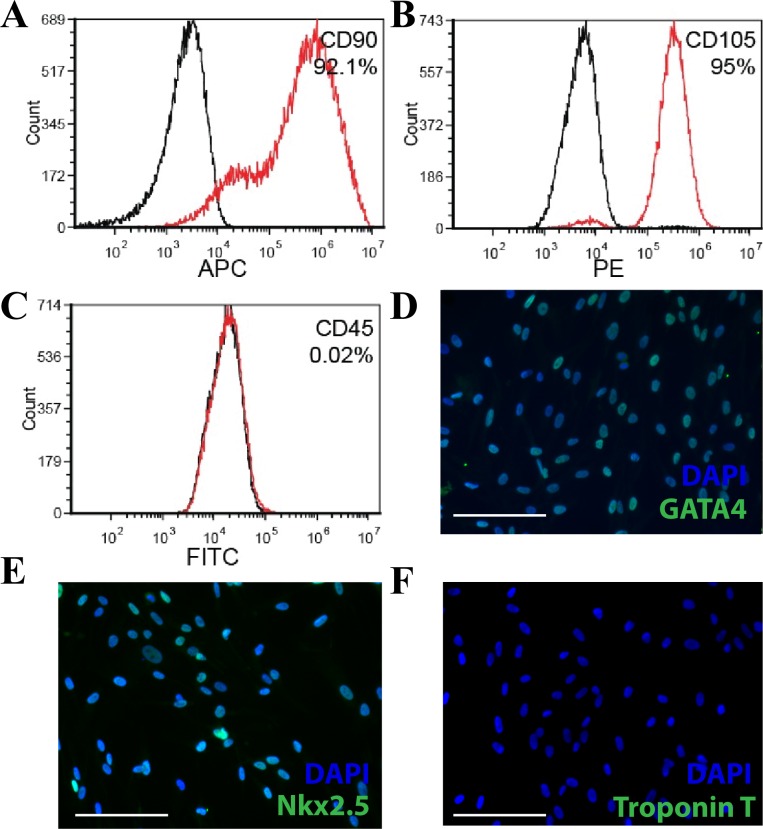
Characterization of human cardiosphere-derived cells (CDCs). (A-C) Representative fluorescence activated cell sorting (FACS) plots of CDCs stained with CD90, CD105 and CD45 (plotted in red) and their respective isotype controls (plotted in black). hCDCs express high levels of mesenchymal markers CD90 and CD105 and are negative for leukocyte common antigen marker, CD45. (D) hCDCs stain positive for cardiac transcription factor GATA4 and Nkx2.5 and negative for Troponin T. Scale bar 200μm.

Right atrial appendage endomyocardial biopsies (n  =  4 patient biopsies; 20–60 mg) were taken from adult patients undergoing on pump coronary artery bypass surgery. CDC cell surface markers were characterized using flow cytometry. Similar to our previously characterized porcine CDCs, human CDCs expressed high levels of mesenchymal markers CD90 and CD105, but were largely CD45 negative. Like previous studies in porcine CDCs [2], the human CDCs also expressed GATA4 and Nkx2.5, markers of early cardiac development, and were negative for cardiac troponin T (**Fig 1**).

**Fig 2:**
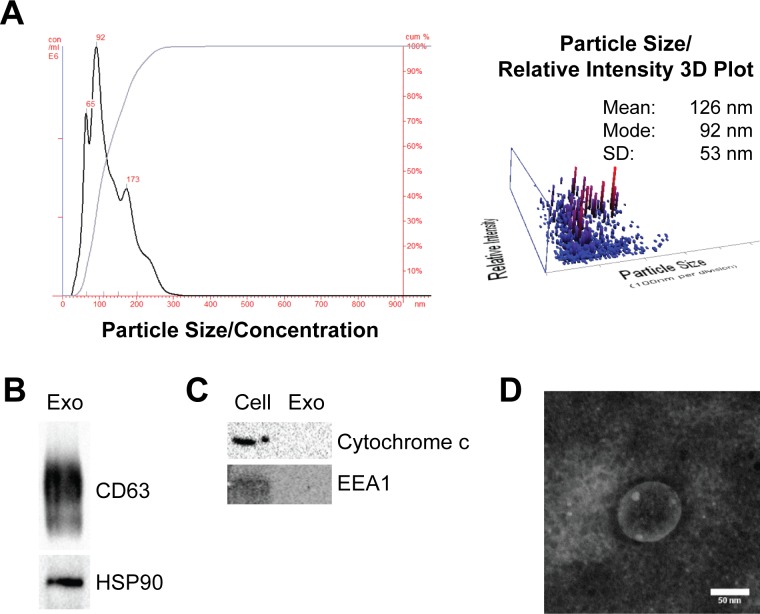
Characterization of CDC-derived exosomes. (A) Nanoparticle analysis of particle distribution profile shows human CDC derived exosomes largely range between 65 and 173nm in diameter. (B) Immunoblot analysis of exosomal markers CD63 and HSP90 in CDC derived exosomes and (C) non-exosomal makers, cytochrome c and EEA1, in exosomes and corresponding CDC whole-cell lysate (15μg/well). (D) Transmission electron microscope image of human CDC-secreted exosome at 100 keV. Negative contrast staining showing typical exosome morphology.

Exosomes were isolated from exosome-free media conditioned for 48 hours by cultured human CDCs or normal human dermal fibroblasts (NHDF), as a control. Particle size analysis and transmission electron microscopy of human CDC exosomes showed a typical size distribution (**Fig 2A and 2D**). In addition, exosomes expressed exosomal marker proteins CD63 and HSP90 and lacked expression of cytochrome c and EEA1 (markers of mitochondria and late endosomes respectively) which were expressed by their parent cells (**Fig 2B and 2C**).

**Fig 3:**
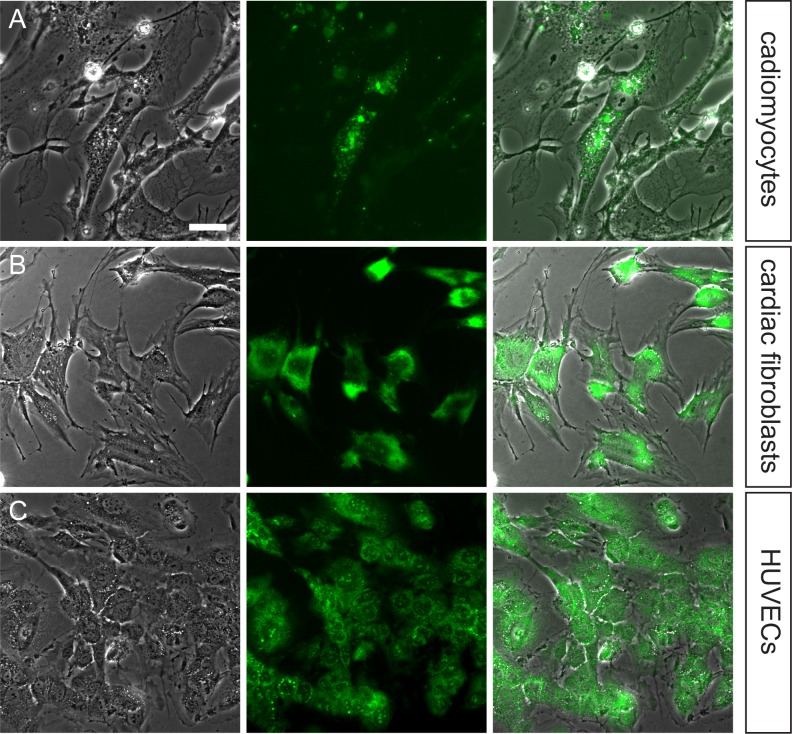
Differential Effect of CDC-derived exosomes are internalized by cardiomyocytes, cardiac fibroblasts and HUVECs. Isolated CDC-derived exosomes labeled with Exo-Red (nucleic acid label) were incubated with (A) cardiomyocytes, (B) cardiac fibroblasts, and (C) HUVECs for 6 hours and imaged to show cellular uptake. The frequency of hCDC-derived exosome uptake was lower for cardiomyocytes than endothelial cells or fibroblasts. Scale bar 50μm.

To determine whether cardiac cell types of interest could internalize exosomes derived from CDCs cardiomyocytes, we incubated cardiac fibroblasts, and HUVECs with fluorescent-labeled exosomes for 6 hours and imaged them with fluorescent microscopy **(Fig 3)**. While each of the target cell types internalized hCDC derived exosomes, there appeared to be a lower frequency of uptake in cardiomyocytes.

### Secretion of exosomes from hCDCs is inhibited by lentiviral nSMase2 shRNAi KD

**Fig 4:**
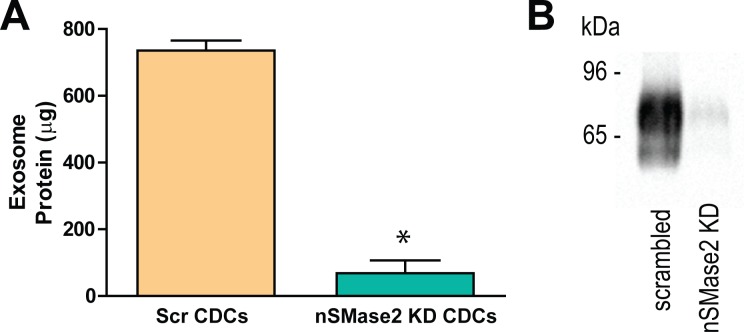
Stable transduced hCDC-nSMase2 lentiviral knockdown lines block exosome secretion. (A) Bradford assay and (B) immunoblot analysis of CD63 of exosomes from CDCs transduced with lentiviral vectors for scrambled or nSMase2-specific shRNAi show nSMase2 knockdown results in significantly less release of exosomes from human CDCs (n = 3). *p<0.01 using two-tailed Student’s t test. Data are presented as mean ± SEM.

To define the functional role of physiological exosome secretion on cardiac cell death and proliferation, we transduced human CDCs with lentiviral shRNA targeting neutral sphingomyelinase 2 (nSMase2), which has been shown to contribute to exosome secretion by triggering the budding of exosomes in MVBs [20]. Consistent with results in other cell lines, knockdown of nSMase2 markedly reduced exosome release relative to scrambled infected control CDCs. Following two days of confluent culture in a T75, P4 CDC exosomal protein content was 735 ± 30 μg in Scr CDCs vs. 68 ± 38 μg in nSMase2 CDCs (mean ± SEM; n = 3; p = 0.0053 using two-tailed Student’s t test) (**Fig 4**). There was no significant difference in the number of CDCs between groups at the time of supernatant collection.

**Fig 5:**
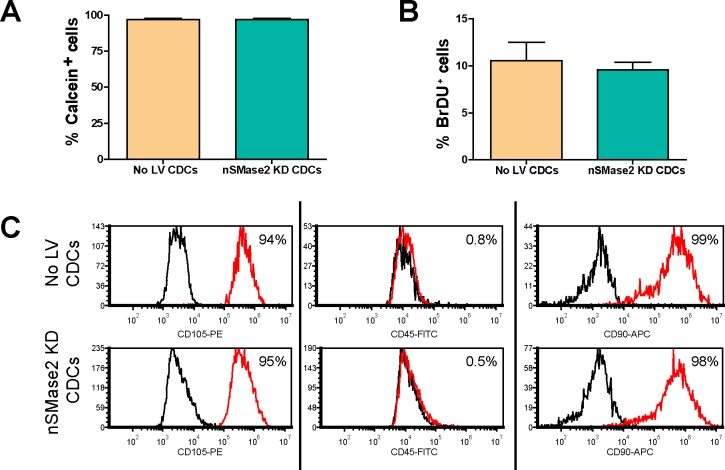
nSMase2 lentiviral integration does not change CDC survival, proliferation or gene expression. Nontransduced CDCs and nSMase2 shRNAi lentiviral infected CDCs show no significant difference in (A) survival assessed with calcien staining, (B) proliferation assessed with BrDU+ pulse or (C) surface marker expression (CD90, CD105 and CD45 plotted in red and their respective isotype controls plotted in black).

Live cell counts with calcein demonstrated that stable CDC lentiviral transduction did not alter the density of live cells. The rate of proliferation in lentiviral infected CDCs was similar to control cells, as assessed by 6 hour BrdU pulse (n = 4 samples, P<0.05) **(Fig 5A and 5B)**. Finally, expression of CD90, CD105, and CD45 as quantified by flow cytometry, was similar in control and lentiviral infected CDCs (**Fig 5C**). These data suggest that neither lentiviral integration nor inhibition of endogenous exosome secretion altered key CDC parameters.

### hCDC exosomes increase endothelial migration, tube formation and branch point complexity without affecting endothelial proliferation

**Fig 6:**
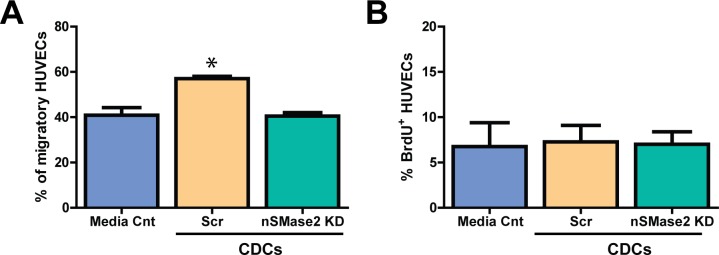
hCDC-derived exosomes promote HUVEC migration without affecting proliferation. (A) The migratory response of HUVECs following hCDC coculture was studied using a 4 hour transwell migration assay. HUVECs cocultured with lentiviral Scrambled control (Scr) CDCs had increased migration as hen compared to endothelial cells cocultured with lentiviral:nSMase2 KD CDCs (57 vs 41%) and media control (n = 3). (B) HUVECs cocultured with Scr CDCs and pulsed with BrdU demonstrated no change in proliferation compared to those cocultured with nSMase2 KD CDCs (% BrdU+ cells/total number of DAPI+ cells; n = 3). *p<0.05 by one-way ANOVA (Tukey’s post hoc test). Data are presented as mean ± SEM.

**Fig 7:**
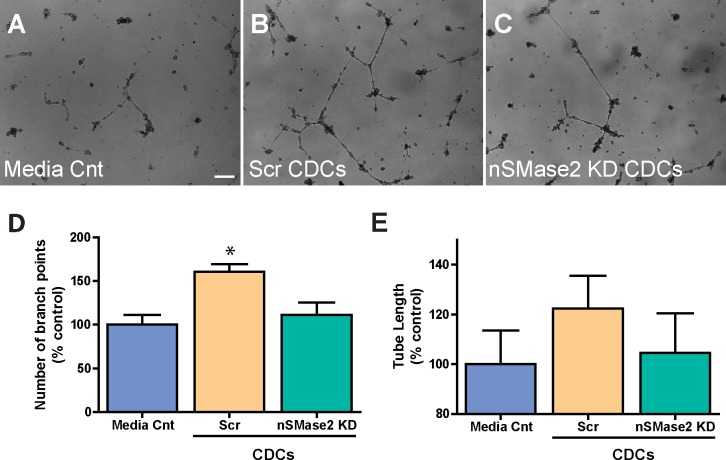
hCDC exosomes stimulate angiogenesis in a HUVEC angiogenesis assay. HUVECs cocultured with Scr CDCs for 24 hours show an increased number of branch points and a trend towards increased tube length as compared with HUVECs cocultured with nSMase2 KD CDCs in a 4 hour matrigel in vitro tube assay (n = 3). Scale bar 200μm. * p<0.001 using one-way AVOVA (Tukey’s post hoc test). Data are presented as mean ± SEM.

To determine the functional effects of exosomes on endothelial cells, we evaluated whether CDC exosome uptake could induce endothelial migration and tube formation. HUVECs cocultured with lentiviral scrambled control (Scr) CDCs showed an increase in migration relative to HUVECs cocultured with nSMase2 KD CDCs (57 ± 1% vs 41 ± 1.5%; mean±SEM) and media control, as assessed by a 4 hour *in vitro* transwell assay (n = 3; p<0.05 using one-way ANOVA with Turkey’s post hoc test) **(Fig 6A)**. In addition, we found that HUVECs cocultured with Scr CDCs for 24 hours showed an increased number of branch points (161 ± 8%) as compared with HUVECs cocultured with nSMase2 KD CDCs (111 ± 14%) and media control (mean ± SEM; n = 3; p<0.001 using one-way ANOVA with Tukey’s post hoc test). Furthermore, although not statistically significant (p = 0.39 using one-way ANOVA with Tukey’s post hoc test), there was a trend towards increased tube length in HUVECs cocultured with Scr CDCs (122 ± 13%) as compared with HUVECs cocultured with nSMase2KD CDCs (105 ± 16%) in a 4 hour matrigel *in vitro* tube assay **(Fig 7)**, indicative of enhanced angiogenesis in HUVECs exposed to CDC secreted exosomes.

Interestingly, there was no statistically significant difference in endothelial cell proliferation (as assessed by 4 hour BrdU pulse) between coculture groups suggesting no effect of exosomes on HUVEC cell division **(Fig 6B)**. In addition, there was no effect of inhibiting nSMase2 on HUVEC viability as assessed by Calcein AM staining (BD Biosciences).

### hCDC exosomes reduce proliferation of human cardiac fibroblasts without affecting cell viability or fibrotic gene expression

**Fig 8:**
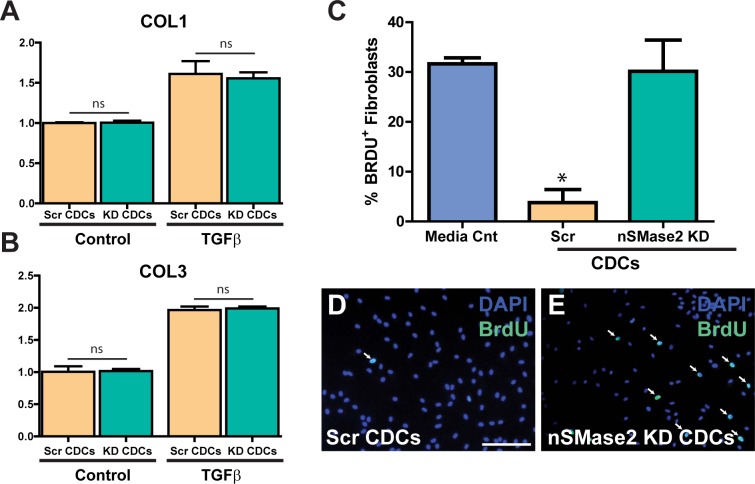
hCDC exosomes reduce proliferation of cardiac fibroblasts without affecting collagen gene expression. (A, B) Human quiesced cardiac fibroblasts cocultured with either Scr CDCs or KD CDCs showed no statistically significant difference in COL1 or COL3 gene expression by qRT-PCR following TGFβ stimulation (n = 3). Data normalized to unstimulated Scr CDCs. (C, D) Cardiac fibroblasts cocultured with Scr CDCs for 24 hours showed a significant reduction in cellular proliferation as assessed by BrdU staining (5 hour pulse) when compared with (C, E) cardiac fibroblasts cocultured with nSMase2 KD CDCs and media control. n = 3. Scale bar 200μm. *p<0.05 using one-way AVOVA (Tukey’s post hoc test). Data are presented as mean ± SEM. White arrows highlight cells that co-stain for DAPI and BrdU.

To investigate the potential effects of exosomes on fibrotic gene expression, we cocultured human primary cardiac fibroblasts with either Scr CDCs or nSMase2 KD CDCs for 24 hours before stimulation with TGF-β to induce a fibrotic response. TGF-β stimulation significantly increased collagen I (COLI) and collagen III (COLIII) mRNA expression by 1.6 ± 0.2 and 2 ± 0.1 fold respectively, as determined by quantitative RT-qPCR (mean ± SEM; n = 3; p<0.05 using two-tailed unpaired Student’s t test), normalized to GAPDH, without demonstrating any significant difference in COLI or COLIII expression between cardiac fibroblasts cocultured with Scr CDCs and nSMase2 KD CDCs (**Fig 8A and 8B**). These observations indicate that CDC exosome secretion and subsequent exosome uptake by cardiac fibroblasts had no effect on collagen gene expression. In contrast, when we looked at cardiac fibroblast proliferation under the same coculture conditions, we saw a significant reduction in proliferation quantified by BrdU uptake following a 5 hour pulse in cells cocultured with Scr CDCs (4% ± 3%) vs. cells cocultured with nSMase2 KD CDCs (30% ± 6%), or media control (32% ± 1%) (**Fig 8C–8E**) (mean ± SEM; n = 3, p<0.05 using one-way ANOVA with Tukey’s post hoc test). There was no change in cell viability between groups as assessed by Calcein AM staining.

### hCDC exosomes decrease cardiomyocyte apoptosis and cause a trend toward increased proliferation of cardiomyocytes *in vitro*

**Fig 9:**
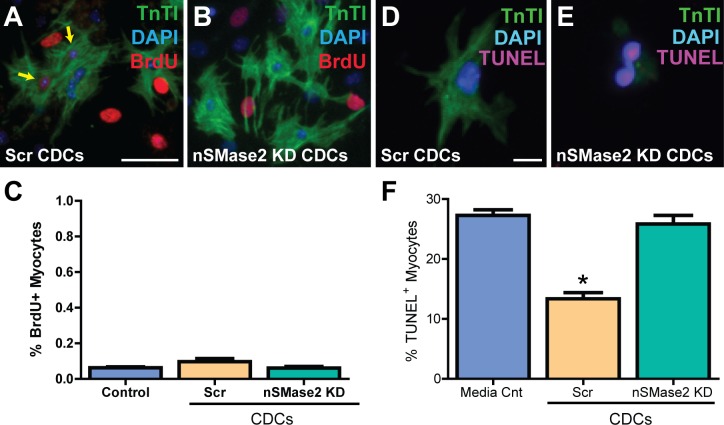
hCDC-derived exosomes significantly decrease cardiomyocyte programed cell death and cause a trend toward increased proliferation of cardiomyocytes *in vitro* at one week in culture. (A, C) Primary mouse neonatal cardiomyocytes (mCM) (P1-3) cocultured with Scr CDCs exhibited a trend toward a higher percentage of BrdU+ cells at one week following a 5 day pulse when compared with (B, C) mCM cocultured with nSMase2 KD CDCs (n = 4; p = 0.19 using one-way ANOVA with Tukey’s post hoc test). Scale bar 50μm. (D, F) mCM cocultured with Scr CDCs exhibited fewer terminal deoxynucleotidyl transferase nick end labeling (TUNEL)-positive nuclei at one week when compared with (E, F) mCM cocultured with nSMase2 KD CDCs and media control (n = 4). The magnitude of the effect of CDC exosomes on myocyte apoptosis was nearly two orders of magnitude larger than proliferation assessed by BrdU over this time frame. Scale bar 10μm. *p<0.001 using one-way AVOVA (Tukey’s post hoc test). Data are presented as mean ± SEM.

To explore the presence of a differential effect of CDC exosomal uptake on cardiomyocyte proliferation vs. apoptosis we cocultured primary mouse neonatal cardiomyocytes (mCMs) with control Scr CDCs and nSMase2 CDCs. mCMs cocultured with Scr CDCs exhibited a trend towards increased proliferation as compared to mCM cocultured with nSMase2 KD CDCs, as evidenced by a higher percentage of BrdU^+^ cells following a 5 day pulse (0.1% ± 0.02% vs. 0.06% ± 0.01%) (mean ± SEM; n = 4, p = 0.19 using one-way ANOVA with Tukey’s post hoc test) (**Fig 9A–9C**). In contrast, endogenous exosomes had a significant effect on preventing myocyte cell death from apoptosis. mCMs cocultured with Scr CDCs exhibited fewer terminal deoxynucleotidyl transferase nick end labeling (TUNEL)-positive nuclei at one week (13% ± 1%) when compared with mCMs cocultured with nSMase2 KD CDCs (26% ± 1%) and media control (27% ± 0.9%) (mean ± SEM; n = 4; p = 0.0006 using one-way ANOVA with Tukey’s post hoc test) (**Fig 9D–9F**).

## Discussion

In this study, we have presented further evidence that CDC-derived exosomes contribute to the modification of the target cardiac tissue milieu and uniquely demonstrated that these effects are mediated by physiological rather than purified and concentrated quantities of exosomes. This is an important observation as it supports the relevance of spontaneous exosome release as a mechanism of CDC mediated cardiac repair *in vivo*.

Pharmacological modulation of endogenous exosome release from CDCs has previously been examined by pre-conditioning cells with GW5869, a frequently used nSMase inhibitor [15]. However, this pharmacological inhibitor is reversible, limiting the conclusions that can be drawn from its use in understanding the role of longer term exosome release in chronic *in vivo* models of cardiac dysfunction. Pharmacological inhibition also lacks specificity, having activity against nSMase1, 2, and 3 [29–31]. In our work, we employed a genetic approach to block CDC exosomal release on a long-term basis using shRNAi to specifically knockdown nSMase2 expression in transduced human CDCs. We demonstrated that similar to macrophages, exosome release from CDCs is dependent on nSMase2. Furthermore, with creation of stable lentiviral cell lines, constitutive inhibition of exosomal release allowed us to examine the role of exosomes in direct co-culture experiments with CDCs and specific target cells. This provides a novel approach to evaluate the role of physiological exosome release on a long-term basis in chronic animal models of cardiovascular disease.

We assessed the contribution of CDC-derived exosomes on specific cell types found within the myocardial cellular milieu that would be exposed to CDC released exosomes *in vivo*. Interestingly, we found that endothelial cell proliferation or endothelial cell death was not affected by coculture of endothelial cells with either Scr CDCs or nSMase2 KD CDCs. Nevertheless, endothelial cells cocultured with control Scr CDCs showed increased migration and tube formation in an *in vitro* angiogenesis assay which was blocked by inhibiting exosome release with knockdown of nSMase2. This stimulation of angiogenic activity is consistent with our previous *in vivo* results showing increased capillary density throughout the heart following intracoronary injection of CDCs in swine with hibernating myocardium[1, 2]. It is also consistent with the increase in capillary density found after focal injection of purified CDC derived exosomes into the border zone of a murine infarction by others [15].

Following acute myocardial infarction, fibroblast proliferation leads to the formation of noncontractile scar tissue [32]. This, in combination with the loss of cardiomyocytes, contributes to long-term systolic myocardial dysfunction [9]. In the infarcted heart, fibroblasts are stimulated by cytokines such as TGF-β which then release pro-fibrotic growth factors which stimulate extracellular matrix production and lead to enhanced fibrosis. Interestingly, we found no significant effect of inhibiting exosome release on basal or TGF- β induced COLI and COLIII expression in cardiac fibroblasts cocultured with CDCs. In contrast, exosomes released from CDCs inhibited fibroblast proliferation and this antiproliferative effect was reversed by inhibiting exosome release with nSMase2 knockdown. These data suggest that while hCDC derived exosomes do not affect fibrotic gene expression, they inhibit fibroblast proliferation which likely contributes to the ability of CDCs to reduce myocardial infarct scar volume in vivo as well as reduce interstitial fibrosis in swine with hibernating myocardium [2]. While fibrotic gene expression has not been previously examined in fibroblasts cocultured with CDCs or purified exosomes, it has been evaluated after treatment with purified exosomes from hypoxic rat ckit^+^ cardiac progenitor cells (CPCs). In contrast to human CDCs-derived exosomes, exposure to rat CPC-derived exosomes has been shown to downregulate COLIII expression in fibroblasts [33]. This observation highlights potential differences in the exosomal cargo of CDCs and CPCs, the two most widely studied cardiac stem cell populations.

We also assessed the effect of physiological CDC-derived exosome release on *in vitro* cardiomyocyte cell death and proliferation. Our data demonstrated significant reductions in apoptosis when cardiomyocytes were cocultured with Scr vs. nSMase2 CDCs. In contrast, there were only insignificant trends toward increased CM proliferation over the relatively brief time frame of our *in vitro* experiments. Furthermore, over a one week time frame, the impact of inhibiting myocyte cell death was 100 fold greater than the denovo generation of myocytes assess using BrdU. These observations suggest that exosomes released from CDCs primarily promote promotes survival of endogenous CMs by inhibiting apoptosis as opposed to stimulating de novo myocyte formation is a subset of surviving CMs. The finding of decreased CM cell death following CDC-derived exosome uptake correlates with the reductions in myocyte apoptosis reported when administering purified CDC derived exosomes immediately after acute myocardial infarction [15]. These findings contrast with the magnitude of *in vitro* cardiomyoyte apoptosis vs. proliferation found by others, where increased CM proliferation and decreased CM apoptosis are approximately balanced. This could be due in part to differences in the cell culture assays as well as species differences between rat and mouse cardiomyocytes [15]. It may also reflect differing time courses over which these end-points are evaluated. For example, when myocyte apoptosis is assessed acutely after infarction *in vivo*, apoptosis rates are high and preserving myocytes by short-term inhibition of apoptosis predominates. In contrast, when evaluated over a period of weeks or months after infarction, apoptosis declines and the cumulative effect of exosome release on a low rate of myocyte proliferation could lead to substantial new myocyte formation. As an example of this, our group has demonstrated substantial cumulative endogenous myocyte proliferation in viable dysfunctional myocardium after global intracoronary infusion of CDCs as well as MSCs.

A limitation of our study is that the results are specifically related to studying CDCs and exosome release in coculture *in vitro*. While our findings are consonant with the effect of CDCs on similar histopathologic end points in swine models of myocardial infarction and hibernating myocardium *in vivo*, additional long-term studies using CDCs with nSMase knocked down in chronic large animal models will be required to establish the impact of inhibiting CDC exosome release *in vivo*. In addition, further work is necessary to elucidate the functionally relevant cargo of CDC-derived exosomes and validate targets *in vivo*.

In summary, our findings support a differential role of exosomes released from CDCs on the major cell types found in the heart. Their effect on cardiac myocytes primarily reflects an anti-apoptotic effect which prevents myocyte death. In contrast, there is primarily an antiproliferative effect of CDC derived exosomes on cardiac fibroblasts with exosome release stimulating capillary reorganization. While CDC derived exosomes do not appear to significantly simulate significant cardiomyocyte proliferation over the time course of our *in vitro* study, a very small pro-proliferative effect could lead to significant new myocyte formation over a longer time frame of weeks or months, as has been demonstrated after intracoronary CDC infusion *in vivo* [2]. Based on our observations, exosomes released by CDCs may have their greatest impact on preventing myocyte death and fibroblast proliferation in the setting of an acute myocardial infarction and account for the favorable short-term effects of intracoronary CDCs infused at the time of myocardial reperfusion [15]. The development of a siRNA approach to effect the long-term inhibition of nSMase and exosome release from CDCs will now facilitate identifying the relative role of exosomes vs. other paracrine factors and direct effects of CDCs on cardiac repair in chronic disease models and help develop translational approaches that afford the best functional outcome from cardiac repair.
